# Circulating Mitochondrial DNA and Inter-Organelle Contact Sites in Aging and Associated Conditions

**DOI:** 10.3390/cells11040675

**Published:** 2022-02-15

**Authors:** Anna Picca, Flora Guerra, Riccardo Calvani, Roberta Romano, Hélio José Coelho-Junior, Francesco P. Damiano, Cecilia Bucci, Emanuele Marzetti

**Affiliations:** 1 Fondazione Policlinico Universitario “Agostino Gemelli” IRCCS, 00168 Rome, Italy; anna.picca@policlinicogemelli.it (A.P.); francescopaolo.damiano@policlinicogemelli.it (F.P.D.); emanuele.marzetti@policlinicogemelli.it (E.M.); 2 Department of Biological and Environmental Sciences and Technologies, Università del Salento, 73100 Lecce, Italy; flora.guerra@unisalento.it (F.G.); roberta.romano@unisalento.it (R.R.); cecilia.bucci@unisalento.it (C.B.); 3 Department of Geriatrics and Orthopedics, Università Cattolica del Sacro Cuore, 00168 Rome, Italy; coelhojunior@hotmail.com.br

**Keywords:** exosomes, extracellular vesicles, inflamm-aging, senescence, mitophagy, mitochondrial damage, mitochondrial dynamics, mitochondrial-derived vesicles, mitochondrial-lysosomal axis, oxidative stress

## Abstract

Mitochondria are primarily involved in cell bioenergetics, regulation of redox homeostasis, and cell death/survival signaling. An immunostimulatory property of mitochondria has also been recognized which is deployed through the extracellular release of entire or portioned organelle and/or mitochondrial DNA (mtDNA) unloading. Dynamic homo- and heterotypic interactions involving mitochondria have been described. Each type of connection has functional implications that eventually optimize mitochondrial activity according to the bioenergetic demands of a specific cell/tissue. Inter-organelle communications may also serve as molecular platforms for the extracellular release of mitochondrial components and subsequent ignition of systemic inflammation. Age-related chronic inflammation (inflamm-aging) has been associated with mitochondrial dysfunction and increased extracellular release of mitochondrial components—in particular, cell-free mtDNA. The close relationship between mitochondrial dysfunction and cellular senescence further supports the central role of mitochondria in the aging process and its related conditions. Here, we provide an overview of (1) the mitochondrial genetic system and the potential routes for generating and releasing mtDNA intermediates; (2) the pro-inflammatory pathways elicited by circulating mtDNA; (3) the participation of inter-organelle contacts to mtDNA homeostasis; and (4) the link of these processes with senescence and age-associated conditions.

## 1. Introduction

Mitochondria are cytoplasmic double-membrane organelles residing within eukaryotic cells. Mitochondria are vestiges of the incorporation of an anaerobic bacterial ancestor into an unicellular eukaryote which occurred over a billion years ago [1]. The endosymbiotic fusion marked the evolution of eukaryotic cells via a serendipitous switch towards aerobic respiration. From an evolutionary perspective, this metabolic change contributed to eukaryote complexity and the development of multicellular life [2]. While initially envisioned as resident and isolated, mitochondria are increasingly recognized as “social” organelles immersed into the cytoplasmic fluid together with other organelles with which they interact and coordinate a plethora of cellular activities [3,4].

Different from other cytoplasmic organelles, mitochondria are semi-autonomous as they possess their own genome, the mitochondrial DNA (mtDNA) [5]. This nucleic acid is a circular double-stranded molecule spanning approximately 16 kb pairs and encodes for 37 genes (13 messenger RNAs, 22 transfer RNAs, and 2 ribosomal RNAs). MtDNA is responsible for the synthesis of hydrophobic protein subunits of the electron transport chain (ETC), the apparatus that enables cellular respiration by coupling oxygen consumption with the generation of a membrane potential at the inner mitochondrial membrane. The latter is the bioenergetic core of the cell via production of adenosine triphosphate (ATP) that fuels most cellular activities. Mitochondria are involved in a number of other vital activities, including regulation of ionic (calcium and iron) levels, hormone synthesis, antioxidant detoxification, iron-sulfur cluster and heme biosynthesis, and programmed cell death [6].

MtDNA exists in hundreds to thousands of copies within the cell. This number, which reflects mitochondria abundance and/or mitochondrial mass, varies according to cell metabolism and in response to several intrinsic and extrinsic stimuli [7]. The large mtDNA copy number compared with the amount needed to support oxidative phosphorylation may reflect the mitochondrial involvement in organelle signaling and/or deployment of immune functions [8].

The recognition of an intrinsic mtDNA immunostimulatory property is a feature that has revolutionized the mitochondrial outlook in the context of several inflammatory conditions [8]. In addition to the pro-inflammatory domains of circulating mtDNA molecules, the formation of non-canonical nucleic acid structures during mtDNA transcription and replication holds unique features that may engage nucleic acid sensors and trigger innate immunity [8].

Although possessing a certain degree of independence, mitochondria are under the control of the nucleus with regard to mtDNA stability and sequence evolution over time [9]. This is made possible by their strategic relocation near the nucleus, whereby mitochondria form contact sites able to generate output signals that can influence the expression of nuclear genes [10].

Dynamic homo- and heterotypic interactions involving mitochondria have been described. Inter-organelle communications are enabled by several processes/structures, including fusion, nanotunnel protrusion, and inter-organelle contact sites [11]. Although the exact mechanisms regulating the formation and the activity of these structures are presently unknown, each type of connection has functional implications that can be regulated to optimize mitochondrial activity to meet the bioenergetic demands of a specific cell/tissue [12].

The analysis of these mitochondrial properties and the molecules involved in such communication may help unveil the complex interactions that mitochondria establish to achieve their own homeostasis. Indeed, mitochondrial metabolism and its alterations have been listed among the nine pillars of the geroscience paradigm, and are a key target amenable for anti-aging interventions [13]. In this regard, the close relationship among mitochondrial dysfunction, inflammation and cellular senescence, and their contribution to aging and age-related conditions confer further priority to the understanding of these processes.

Here, we provide an overview of (1) the mitochondrial genetic system and the potential routes for generating and releasing mtDNA intermediates; (2) the contribution of circulating mtDNA to inflammation; (3) the participation of inter-organelle contact sites to mtDNA homeostasis; and (4) the link of these processes with senescence and age-associated conditions.

## 2. Mitochondrial Genetics: MtDNA Transcription, Replication, and the Generation of Non-Canonical Structures

The transcription of mtDNA guides the synthesis of a subset of hydrophobic ETC complex subunits through mtDNA-encoded ribosomes (12S and 16S) and 22 tRNAs [14]. Hydrophyilic ETC subunits and proteins involved in mtDNA transcription, translation, replication, and maintenance are nuclear-encoded and are co- or post-translationally imported into the organelle [7,15].

A large non-coding arc of mtDNA, the so-called displacement-loop (D-loop) region, harbors both the heavy- (HSP) and the light-strand promoter (LSP) of mtDNA transcription, along with the origin of heavy-strand replication (OH) and conserved *cis*-acting elements. Hence, the D-loop is a major regulatory site of mtDNA activity and synthesis [16].

**Figure 1:**
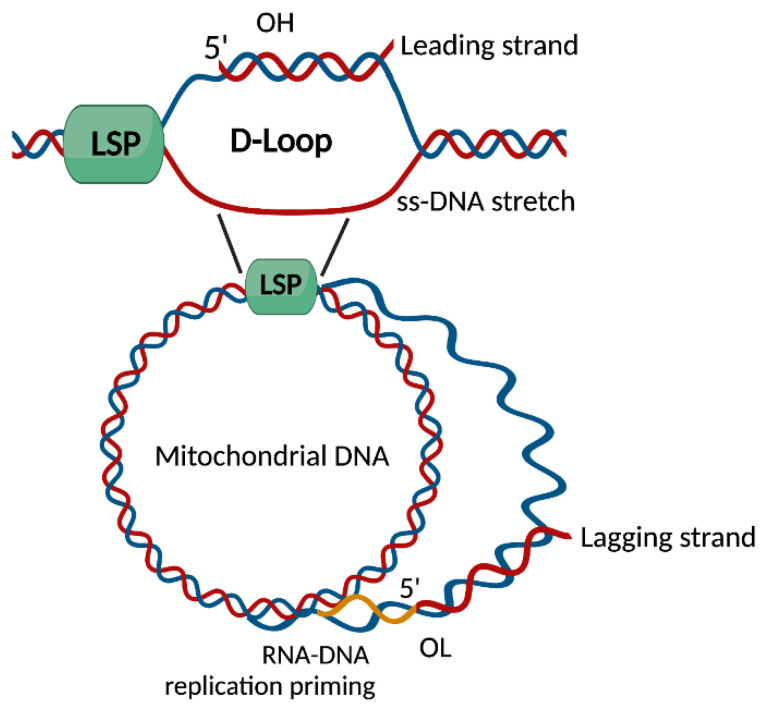
Molecular events of mtDNA expression and replication. D-loop, displacement-loop; LSP, light-strand promoter; OH, origin of heavy-strand replication; OL, origin of light-strand replication; ss-DNA, single-stranded DNA. Created with BioRender.com, accessed on 21 October 2021.

The expression of mtDNA genes starts at both promoters and begins with the transcription of RNA primary transcripts (for both mtDNA strands) that are almost full-length and are processed into mature mRNAs by specific RNases. Almost synchronously, the transcripts generated by the LSP are used as primers for mtDNA replication of the leading strand that follows a replicative asymmetric mode. The termination of transcription and/or specific RNA processing occurs downstream LSP, and strand extension by the mtDNA polymerase γ (Pol γ) generates the 3′ end of this mtDNA strand. Following Pol γ-guided DNA synthesis, the enzyme activity becomes stalled or terminated approximately 1 kb past the LSP. The newly synthesized mtDNA strand remains bound to the template and forms a stable three-stranded D-loop structure. This latter is a hallmark of the mammalian mtDNA replicating structure with uncertain biological relevance [14,16] (Figure 1). MtDNA synthesis downstream from the 3′ end of the D-loop region is mandatory for mtDNA replication, which requires priming of the lagging strand at multiple mtDNA sites. A major replication starter is the origin of light-strand replication (OL), which is located ~12 kb away from the OH and enables asynchronous replication of the leading and lagging strands. As a result of these events, large stretches of single-stranded DNA (ss-DNA) and RNA–DNA hybrids originate and persist as intermediates of mtDNA synthesis. Other modes of mtDNA replication have been described and involve a “bootlace” mechanism following the incorporation of processed mtDNA transcripts [17] (Figure 1).

While an intrinsic immunostimulatory property has been recognized in the bacterial-like hypomethylated CpG motifs of the mtDNA molecule [18], the generation of non-canonical nucleic acids structures during mtDNA transcription and replication has also been suggested to be sensed by and trigger innate immunity by engaging nucleic acid sensors [8]. In particular, ssDNA, RNA–DNA hybrids, and mtDNA-derived higher-order nucleic acid structures, including triplexes, R-loops, and four-way junctions, are recognized by the innate immunity via the cyclic guanosine monophosphate–adenosine monophosphate synthase (cGAS) and other pattern-recognition receptors (PRRs) [8]. These supramolecular structures are able to hijack the repair systems and have gained much attention as mechanisms that may bridge the molecular routes regulating mitochondrial biogenesis with those controlling tissue homeostasis [19].

## 3. Mitochondrial DNA Mutations and Diseases

A wide range of mtDNA mutations and polymorphisms have been identified in several pathological conditions (i.e., chronic progressive external ophthalmoplegia, Kearns–Sayre syndrome, Leber hereditary optic neuropathy (LHON), mitochondrial encephalopathy, lactic acidosis, stroke-like episodes, myoclonus, epilepsy, ragged-red fibers, and neurogenic weakness with ataxia and retinitis pigmentosa) (reviewed in [20]). Of note, both mutated and wild-type mtDNA allele variants can co-exist in the same individual, a condition referred to as heteroplasmy, which explains the wide spectrum of disease severity [20].

A high proportion of mutated mtDNA molecules must be harbored by the cell to impact oxidative phosphorylation and ATP production (threshold effect) [21]. Therefore, individuals inheriting a high proportion of mtDNA heteroplasmy are more prone to severe disease than those with low levels. However, families affected by diseases transmitting only mutated mtDNA (homoplasmy) (i.e., LHON) show very low penetrance. Hence, factors other than genetics, including physiological and environmental conditions, have an impact on disease etiology and penetrance [22]. Moreover, a specific mtDNA polymorphic variation (i.e., population haplogroup) and, in particular, the European haplogroup J have been identified in several LHON families [23], supporting a role for the genetic background in the clinical expression of the disorder [24]. Common haplogroups have also been associated with the risk of developing neurodegeneration, (i.e., Parkinson’s disease [25] and Alzheimer’s disease [26]), and other late-onset disorders, including type II diabetes [27,28].

Acquired mtDNA mutations have been identified in tissues and organs of people with late-onset disorders [29,30]. In particular, combinations of point mutations and large-scale mtDNA deletions can accumulate at different rates in the cells and undergo clonal expansion over the life-course until reaching critical levels and affecting cellular bioenergetics. The generation of preclinical models bearing these mutations has allowed for the establishment of a causal link between the clonal expansion of mtDNA mutations and age-related conditions [31,32], as well as their role as drivers of aging itself. The accumulation of mtDNA mutations may contribute to the appearance of aging phenotypes through mechanisms involved in tumorigenesis and cellular senescence [33,34]. Indeed, mtDNA mutations promote tumor growth via metabolic remodeling which triggers cellular senescence as an oncosuppressive response. Over time, the accumulation of mtDNA mutations increases the burden of senescent cells in the body and contributes to the development of aging phenotypes [35].

MtDNA heteroplasmy seems to occur more frequently than previously thought [36,37], and the high mutational load observed in older age may result from life-long clonally expanded mtDNA mutations that were likely inherited at a very low level of heteroplasmy at birth [38]. While the exact mechanisms regulating mtDNA mutation inheritance and penetrance are largely unknown, several lines of evidence indicate that mtDNA manipulation may represent a promising route for preventing and treating diseases for which mtDNA mutations play a key role.

The involvement of mtDNA in pathological conditions has largely been investigated in relation to inherited and/or acquired mutations in resident mtDNA. However, there is also evidence for a possible role of mtDNA displacement into the circulation in the pathogenesis of several conditions. In particular, a pattern of pro-inflammatory mediators pertaining to innate immune responses, including tumor necrosis factor alpha (TNF-α) and interferons (IFNs), have also been indicated to link cellular senescence with chronic low-grade inflammation [39]. In the next sections, the mechanisms of mtDNA release and the inflammatory signaling pathways elicited by circulating mtDNA molecules during aging and associated conditions are described.

## 4. Mitochondrial DNA: A Signaling Molecule beyond Organelle Boundaries

The proximity of the mitochondrial genome to the ETC, a major intracellular source of reactive oxygen species (ROS), exposes mtDNA to oxidative damage, thus making mtDNA highly polymorphic and subject to a high mutational rate. These mutations can interfere with ETC assembly and, hence, contribute to mitochondrial dysfunction via impairment of ATP production, dispersion of transmembrane potential, and increase of ROS production [40]. These events culminate in oxidative damage to cellular structures.

The possibility that mtDNA, along with other organellar components, may translocate and signal into the cytosol or at the extracellular level was not fully appreciated until recently. Indeed, mtDNA has been shown to be released into the cytosol in a dose-dependent manner under oxidative stress induced by lipopolysaccharide [41]. To reach out the cytosol, the mitochondrial genome must cross the inner and the outer mitochondrial membranes. This process may be facilitated by the opening of mitochondrial permeability transition pores [42]. Apoptosis is one of the mechanisms promoting the formation of these macro-pores for mtDNA escape. Indeed, activation of the pro-apoptotic proteins BAK and BAX form gateway structures for mtDNA herniation and release into the cytosol [43]. In this scenario, the integrity of mtDNA and the morphology of the mitochondrial membrane are preserved as opposed to necrosis that involves mtDNA rupture [44]. Therefore, intact mtDNA is released from the mitochondrial matrix into the cytosol during apoptosis, whereas necrosis may release mtDNA fragments outside the cells as circulating cell-free (ccf)-mtDNA. Indeed, ccf-mtDNA has been detected in the extracellular fluid of necrotic cells in the setting of acute tissue injuries, such as trauma, acute myocardial infarction, and sepsis [45].

Cells may also enact other forms of mitochondrial outer membrane permeabilization (MOMP) that are characterized by partial depolarization [46]. MOMP is triggered in the setting of mild stressors that favor the oligomerization of the voltage-dependent anion channel proteins (VDAC1 and VDAC3) and lead to pore formations that allow mtDNA fragments to reach the cytosol [47]. In this case, inner mitochondrial membrane permeabilization occurs through yet unknown mechanisms which may involve the mitochondrial permeability transition pore [48].

Ccf-mtDNA is not necessarily membrane-free. Indeed, the human plasma also contains intact cell-free mitochondria [49]. In addition, ccf-mtDNA has been retrieved within extracellular vesicles (EVs), a set of small lipid membrane vesicles of ~30–400 nm of diameter that are released by several cell types. Different types of EVs have been identified according to their surface characteristics and biogenesis. The majority of EVs are exosomes, microvesicles, and apoptotic bodies, although current isolation techniques make it difficult to differentiate the various subtypes. Exosomes are released through the fusion of a multivesicular body with the plasma membrane. Since these specific subtypes of EVs emerge from the endo-lysosomal pathway, they are very informative of the intracellular degradative routes regulating cellular quality control processes. Microvesicles, instead, are formed through pinching off of the plasma membrane, and apoptotic bodies are released during apoptosis [50,51]. Proteins, lipids, and nucleic acids are delivered to target cells by EVs [52,53,54] and the pathophysiological status of the originating cell influences the composition of newly-formed EVs [55]. MtDNA fragments have been identified within exosomes released by astrocytes and myoblasts [56,57]. EVs derived from mesenchymal stem cells and astrocytes in response to oxidative stress and containing mitochondrial components in addition to mtDNA have also been described [58,59]. Nevertheless, the mechanisms regulating the loading of mitochondrial constituents into EVs and their role/signaling outside the cells require further investigation.

The whole mitochondrial genome has been identified in circulating EVs isolated from patients with metastatic breast cancer resistant to hormonal therapy, and a role in cancer resistance has been proposed for this horizontal mtDNA transfer [60]. In particular, the acquisition of mtDNA through EVs was shown to restore oxidative phosphorylation in cancer stem-like cells [60]. Moreover, cancer cells producing higher levels of mtDNA-enriched EVs were also able to induce faster metabolic reprogramming in response to oxidative stress and contribute to hormonal therapy resistance [60]. However, few data are available on exosome characteristics and signaling in aging and associated conditions. For instance, circulating MDVs have been identified in older adults with physical frailty and sarcopenia [61,62] and in patients with Parkinson’s disease [63]. The observation that EVs derived from mesenchymal stem cell were able to attenuate mitochondrial damage and inflammation by stabilizing mitochondrial DNA may indicate that these EVs hold signaling roles and may represent pivotal mediators in conditions characterized by dysfunctional mitochondria [64]. The recognition of mitochondrial disfunction among the pillars of aging makes the characterization of mitochondrial-derived vesicles (MDVs) a relevant mechanism to be investigated to identify biomarkers that may be informative on aging and related phenomena.

Mitochondrial markers were also identified in larger platelet-derived EVs using flow cytometry [65] and visualized by electron microscopy analysis within EVs released by mesenchymal stem cells as an attempt to outsource mitophagy [58]. Similarly, mitochondrial-derived EVs have been identified among the EVs produced by activated monocytes to stimulate type I IFN and TNF responses in endothelial cells [66]. Finally, EVs can transfer mtDNA from T lymphocytes to dendritic cells [67] to trigger an inflammatory response via the toll-like receptor-9 (TLR-9)−nuclear factor kappa B (NF-κB) pathway in patients with heart failure [56].

## 5. Circulating Cell-Free MtDNA: A Trigger of Inflammation

Ccf-mtDNA can be present either as double-stranded short (<1 kb) or long (up to 21 kb) fragments. Whether these mtDNA fractions have a functional role similar to mtDNA molecules released by differentiated cell populations is currently unknown [68].

Ccf-mtDNA acts as a damage-associated molecular pattern (DAMP) and is able to trigger inflammation, coagulation, and immunity, as well as to induce cell death and tissue damage [69]. MtDNA is immunogenic because of its bacterial ancestor. Although DNA methyltransferases are present within mitochondria [70,71], mtDNA contains many unmethylated CpG motifs [72] that can trigger inflammation through the activation of pattern recognition receptors (PRRs) such as TLR9 [72,73,74,75]. These PRRs are differentially expressed in tissues and cell types, and the pro-inflammatory effects of mtDNA can be enhanced by oxidative modifications [76]. Immune cells such as monocytes, macrophages, plasmacytoid dendritic cells, and B lymphocytes [77] express TRL9, as well as other cells such as hepatocytes, epithelial cells, and cardiomyocytes [73,78,79].

MtDNA triggers inflammation through activation of TRL9 within the endolysosomal compartment [72,80]. TRL9, in turn, activates the adaptor myeloid differentiation primary response protein 88 (MyD88)/mitogen-activated protein kinases (MAPKs)/NF-κB or IFN regulatory factor 7 (IRF7) pathway [81,82], with subsequent production pro-inflammatory cytokines and adhesion molecules to enhance leukocyte differentiation and extravasation into tissues [82]. High levels of ccf-mtDNA have been associated with the development of cardiovascular disease, including atherosclerosis, hypertension, acute myocardial infarction, and heart failure via triggering the TLR9-dependent inflammatory pathway [41,77,83,84].

The inflammasome, a cytosolic multiprotein machinery mainly expressed in immune cells such as macrophages, is another target of circulating mtDNA. This complex is constituted by four receptors, including nucleotide-binding oligomerization domain (NOD), leucine-rich repeat (LRR) receptor kinase, and (NOD)-like receptor family pyrin domain containing protein 1 (NLRP1) and NLRP3, NLR family CARD domain-containing protein 4 (NLRC4), and absent in melanoma 2 (AIM2) [85]. Intact and oxidized mtDNA can promote inflammation via inflammasome activation by binding to the NLRC4 and NLRP3 complexes, respectively [86]. During myocardial ischemia/reperfusion injury, cardiac fibroblasts show upregulation of NLRP3 inflammasome [87]. Moreover, in people with type 2 diabetes, circulating mtDNA is sensed and recognized by AIM2 inflammasome determining an increased production of interleukin (IL)-1β and 18 by macrophages [88,89].

An additional component of the innate immune system is the cGAS-stimulator of interferon genes (STING) DNA-sensing pathway. cGAS binds mtDNA and recruits STING to induce IRF-3 phosphorylation via TANK-binding kinase (TBK). The production of type I and type III IFNs (b and k1) and IFN-stimulated nuclear gene product are induced by IRF-3 [90]. Conversely, the cleavage of cGAS and the downstream transcription factor IRF-3 via apoptotic caspases may act as a non-inflammatory mechanism of cell demise by impairing cGAS sensing of mtDNA [91,92,93].

**Figure 2:**
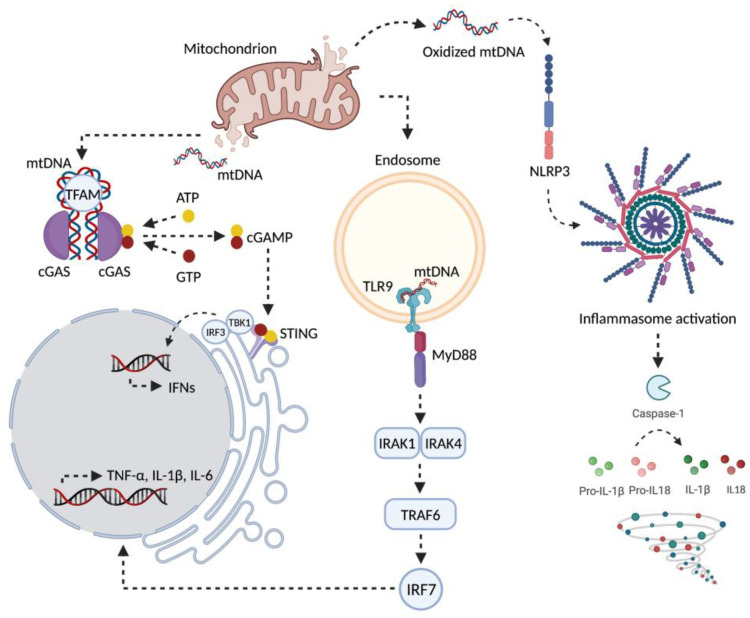
Schematic representation of major signaling pathways through which the displacement of mitochondrial components can trigger inflammation. A decline in the efficiency mitochondrial quality control processes may lead to the intracellular accrual of oxidized components, including mtDNA, that further engulf the mitophagy machinery. These debris can be cleared by the cell along alternative non-degradative routes that release mitochondrial-derived components into the cytoplasm or the extracellular compartment. Displaced mitochondrial-derived components can be recognized as damage-associated molecular patters and trigger inflammation by activating three distinct signaling pathways via the interaction with (1) cytosolic cyclic GMP-AMP synthase (cGAS)-stimulator of interferon genes (STING) DNA-sensing system; (2) toll-like receptors (TLRs); (3) nucleotide-binding oligomerization domain (NOD)-like receptor family pyrin domain containing 3 (NLRP3) inflammasome. ATP, Adenosine triphosphate; cGAMP, Cyclic guanosine monophosphate–adenosine monophosphate; GTP, guanosine triphosphate; IFNs, interferons; IL, interleukin; IRAK, interleukin 1 receptor associated kinase; IRF, interferon regulatory factor; mtDNA, mitochondrial DNA; MyD88, Myeloid differentiation primary response 88; TBK1, TANK-binding kinase 1; TFAM, mitochondrial transcription factor A; TNF-α, tumor necrosis factor-α; TRAF, TNF Receptor Associated Factor 6. Created with BioRender.com, accessed on 18 January 2022.

Finally, DNA PRRs are also activated by intermediates of mtDNA replication and transcription. Indeed, mtRNA−DNA hybrids that form during transcription, long stretches of ss-DNA, and R-loops containing RNA−DNA hybrids with a non-template ss-DNA are sensed and recognized by cGAS [73] (Figure 2). The identification of high levels of pro-inflammatory mediators pertaining to innate immune responses in cellular senescence and chronic low-grade inflammation lends further support to a role of these mtDNA intermediates in triggering PRR-mediated responses [39]. However, their specific involvement in age-related conditions and senescence is currently unknown.

## 6. How Mitochondria “Socialize”: Mitochondrial Contact Sites

Cellular organelles are not isolated but establish physical interactions with one another. Communications between mitochondria and other cellular components are vital for deploying their functions and their dynamics change with age [94]. As such, mitochondrial contacts are crucial molecular platforms contributing to age-associated mitochondrial dysfunction and gateways for mtDNA release and signaling.

### 6.1. Mitochondria−Endoplasmic Reticulum

**Figure 3:**
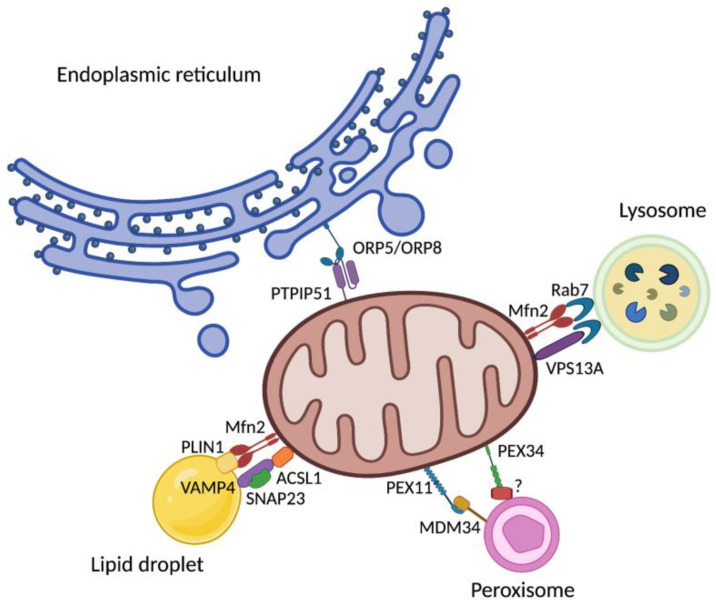
Schematic representation of mitochondrial contact sites. To coordinate all the activities and achieve homeostasis, mitochondria use molecular platforms through which establish contacts with the endoplasmic reticulum, lysosome, peroxisome, and lipid droplet. ACSL1, acyl-CoA synthase long chain family member 1; Mfn2, mitofusin 2; MDM34, Mitochondrial distribution and morphology protein 34; ORP, oxysterol-binding protein-related proteins; PEX, peroxisomal integral membrane protein; PLIN1, peripilin 1; PTPIP51, protein tyrosine phosphatase interacting protein 51; RAB7, Ras-related in brain 7; SNAP23, synaptosomal-associated protein 23; VAMP4, vesicle-associated membrane protein 4; VPS13A, Vacuolar Protein Sorting 13 Homolog A; Created with BioRender.com, accessed on 21 January 2022.

Physical interactions between mitochondria and the endoplasmic reticulum (ER) membranes are referred to as mitochondria−ER contacts (MERCs) (Figure 3). Several aspects of mitochondrial functions, such as mitochondrial dynamics, calcium homeostasis and mitophagy are influenced by MERCs [95]. Due to their implication in all these activities, and especially in the modulation of redox signaling, altered MERCs have been associated with aging and related disorders [96].

Mitochondrial dynamics consist of fission and fusion events that allow shaping mitochondrial morphology from fragmented to filamentous organelles. MERCs are rich in proteins involved in fission and fusion. Mitochondrial fission is favored by ER tubules that interact with mitochondria, defining fission sites and enhancing dynamin-related protein 1 (DRP1) functions [97]. DRP1 is recruited by the mitochondrial fission 1 protein (FIS1) and the mitochondrial fission factor (MFF) that can also localize at the ER, thus representing a platform for DRP1 oligomerization [98]. Moreover, ER-localized inverted formin 2 (INF2) stimulates actin polymerization at ER-mitochondrial contact sites, increase calcium efflux from the ER to the mitochondria, and initiate mitochondrial membrane constriction via myosin, favoring DRP1 oligomerization and DRP1-mediated fission [99]. Less is known about the reason why these proteins are shared by ER and mitochondria. One possibility is that ER behaves as a regulator of mitochondrial dynamics being responsible for sequestration of these proteins in case of excessive fission or initiation of mitochondrial membrane constriction if fusion is too low [94].

MERCs are also involved in mitochondrial fusion. Key proteins in this process are mitofusin (Mfn) 1 and 2. Mfn2 is one of the first identified proteins localized at MERCs, while Mfn1 does not localize to ER [100,101]. Mechanisms by which Mfn2 regulates MERCs are still unclear as it was demonstrated that MERCs formation can be both promoted and inhibited by Mfn2 [102,103]. Aging is associated with reduced ER-mitochondria contact sites, thus having a negative impact on mitochondrial dynamics and several other processes regulated by the two organelles [104].

Intracellular calcium is stored mostly in the ER and mitochondria, and MERCs represent sites where calcium is exchanged between the two organelles [95]. Calcium can exit from the ER through the 1,4,5-trisphosphate receptor (IP3R) and can enter mitochondria via the mitochondrial calcium uniporter (MCU). Moreover, mitochondrial permeability transition pore and ER sarco/endoplasmic reticulum Ca2^+^-ATPase (SERCA) enable calcium release from mitochondria and uptake by the ER, respectively [105].

Mitochondrial fragmentation observed in aged cells can also be explained by an altered calcium exchange between the ER and mitochondria, with increased calcium release from the ER followed by a decreased uptake by mitochondria. Indeed, the mitochondrial localization of DRP1 is stimulated by calcium derived from the ER [106]. Mitochondrial calcium uptake is also regulated by oxysterol-binding protein-related protein 5 (ORP5), localized at MERCs [107]. This protein is also involved in regulating mitochondrial function. Indeed, at ER-mitochondria contact sites, ORP5 and ORP8 interact with the protein tyrosine phosphatase interacting protein 51 (PTPIP51), localized on the outer mitochondrial membrane, and defects in mitochondrial morphology and respiratory functions are observed following ORP5/ORP8 deletion [107]. The interaction between these proteins enable the transfer of phosphatidylserine from the ER to mitochondria, where it is converted in phosphatidylethanolamine (PE) which is important for mitochondrial structure and function [107]. Therefore, the lipid composition of ER−mitochondria interface can be modulated by MERCs, which also influences other processes—such as autophagy—that are stimulated by increased levels of PE [108]. The selective degradation of mitochondria by mitophagy is influenced by ER−mitochondrial contacts. Indeed, calnexin on the ER membrane interacts with FUN14 domain-containing 1 (FUNDC1), localized at the mitochondria, thereby stimulating the recruitment of DRP1 during mitophagy in order to initiate mitochondrial fission [109]. Therefore, mitophagy is reduced with age also as a consequence of a decrease in ER−mitochondria contacts [110].

Mitochondrial morphology is influenced by a process that occurs specifically in the ER, known as ER-specific unfolded protein response (ER−UPR) [111]. Unfolded proteins activate the protein kinase RNA-like endoplasmic reticulum kinase (PERK) which, in turn, activates eIF2α, that causes a reduction of protein translation [112]. Mitochondrial morphology is regulated by this mechanism that favors an increased ATP production to enable the cell to cope with ER−UPR, a process named stress-induced mitochondrial hyperfusion (SIMH) [113].

In aging cells, impaired ER−mitochondria contact sites cause accumulation of misfolded protein at the ER−mitochondrial interface [104]. Therefore, it is not surprising that age-associated disorders are characterized by accumulation of toxic proteins at the ER−mitochondria interface, having negative effects on calcium uptake, autophagy, mitochondrial respiration, and ROS production [114].

### 6.2. Mitochondria−Lysosomes

The existence of a connection between mitochondria and lysosomes has become clear since the observation that alterations in lysosomes, often seen in neurodegenerative diseases, were reflected by impaired mitochondrial networks [115]. The first observations came from experiments in yeast cells where decreased vacuolar acidification and altered mitochondrial dynamics were detected [116]. In the same paper, the authors demonstrated that mitochondrial fragmentation was prevented by the expression of the subunit A of the yeast V-ATPase V1 domain. The treatment with the inhibitor of V-ATPase concanamycin A did not rescue mitochondrial fragmentation, indicating that this was strictly dependent on V-ATPase activity [116].

The close relationship between mitochondria and lysosomes is mediated by two transcription factors, TFAM and transcription factor EB (TFEB). The first is fundamental for mtDNA transcription and replication, and, therefore, mitochondrial biogenesis [117]. Likewise, TFEB is crucial for lysosomal biogenesis [118]. A recent report showed that TFAM-deficient cells are characterized by high mitochondrial fragmentation and dysfunction. In these cells, the nuclear translocation of TFEB activates the mitochondrial master-regulator peroxisome proliferator-activated receptor-gamma coactivator 1 alpha (PGC-1α) leading to co-regulated lysosomal and mitochondrial biogenesis [119,120].

Mitochondria and lysosomes have also physical interactions (Figure 3), as demonstrated by Wong and co-workers [121]. These contacts are mediated by active GTP-bound lysosomal Ras-related in brain (RAB) 7, while FIS1, through recruiting Tre-2/Bub2/Cdc16 member 15 (TBC1D15) to mitochondria, determines the separation of two organelles, stimulating RAB7 GTPase activity [121]. Contacts are important for mitochondrial dynamics since the same group demonstrated that mitochondrial fission was decreased by the expression of a constitutively active mutant of RAB7 (RAB7Q67L), increasing mitochondria-lysosomes contacts [121]. The formation of these contacts is totally independent of metabolite transfer, autophagosome biogenesis or mitophagy, as demonstrated by the fact that mitochondria-lysosomes do not show expression of autophagosomal markers [121], nor are they influenced by genetic ablation of autophagy receptors [122].

Mitophagy is not only influenced by the ER−mitochondria, but also by mitochondria−lysosome contacts. The interaction between TBC1D15, microtubule-associated protein 1 light chain 3 (LC3), and FIS1 is pivotal for regulating RAB7 activity and the formation of the isolation membrane that engulfs damaged mitochondria. Instead, LC3-tagged phagosomes without cargo orientation appear when TBC1D15 is depleted or its RABGAP activity is inhibited [123]. These findings indicate that the formation of the isolation membrane requires RAB7 activation, while the detachment of LC3-positive membranes from microtubules needs RAB7 inactivation [124].

During aging, a progressive reduction of mitophagy occurs, thereby leading to the accumulation of damaged mitochondria with a detrimental effect on cellular homeostasis and with particularly negative effects for long-lived cells, more sensitive to this phenomenon [125]. Recently, a new mechanism for the elimination of damaged mitochondria was identified and described to operate via MDVs [126,127].

The molecular mechanisms that coordinate the formation of MDVs are still poorly understood. One possibility is represented by the accumulation of proteins near mitochondrial membranes, under oxidative stress conditions. In response to protein aggregation, mitochondrial membrane originate curvatures and produce a vesicle [128]. This latter can reach the late endosomes/multivesicular bodies (MVBs) [129] or peroxisomes for cargo detoxification [130]. However, MDVs from mildly damaged mitochondria can fuse with MVBs and be secreted as exosomes, which may represent an indirect evidence of mitochondria/endolysosomal system crosstalk [60,63].

### 6.3. Mitochondria−Peroxisomes

As mentioned above, mitochondria and peroxisomes are in communication through MDVs [130]. Interestingly, they also show physical interaction (Figure 3). Indeed, the peroxisome membrane elongation and fission protein peroxisomal integral membrane protein 11 (PEX11) interacts with mitochondrial distribution and morphology protein 34 (MDM34), localized on mitochondria [131]. Moreover, peroxisomes share the fission machinery with mitochondria. DRP1 is required for the formation of a constriction ring that allows peroxisomal fission [132]. DRP1 is recruited at the constriction sites by PEX11β which does not have scission activity, but peroxisomes appear enlarged and elongated following its ablation, thus indicating that it is essential for peroxisomal fission through modulation of DRP1 activity [131,133,134].

The mechanisms of mitochondrial-peroxisomal communication are still under investigation, but, given the importance of peroxisomes and mitochondria in lipid metabolism, ROS signaling, and protein exchange, they may have a role in aging [135].

### 6.4. Mitochondria−Lipid Droplets

Though for long time considered just an inert cytoplasmatic inclusion of fat, in recent years lipid droplets (LDs) have begun to be considered as full-fledged organelles with several functions [136]. Every eukaryotic cell contains LDs and their main function is the storage of fatty acids in the form of neutral lipids, mostly triacylglycerols and sterol esters, and hydrolysis [137]. To carry out this function, they possess a unique structure encompassing a core of neutral lipids surrounded by a phospholipid monolayer [138]. Several proteins, such as perilipins (PLINs), are associated with the monolayer and are fundamental for LD functions and dynamics [139,140].

The size of LDs changes depending on nutrient conditions, with an enlargement in the case of fatty acid abundance and shrinking during starvation, when fatty acids are used for energy production through β-oxidation [141]. Cellular homeostasis is guaranteed by these organelles, protecting the cells from lipotoxicity caused by excessive fatty acid accumulation [136]. Indeed, dysregulation of lipid metabolism is associated with several diseases, many of which are also characterized by an increase in LD abundance [142]. In this context, LDs have gained attention for their potential role in cancer and neurodegeneration. For instance, a form of Charcot–Marie–Tooth type 2 (CMT2) disease, an axonal neuropathy affecting the peripheral nervous system, is caused by a missense mutation in the diacylglycerol O-Acyltransferase 2 (DGAT2Y223H), an enzyme involved in the synthesis of triglycerides [143]. Moreover, in CMT2 type 2 B (CMT2B), caused by mutations in RAB7 gene, an accumulation of LDs was observed in the cytoplasm, further highlighting the role of altered LD dynamics in neurodegeneration [144,145].

Originally it was thought that inter-organelle contact sites only concerned subcellular compartments with a phospholipid bilayer. However, inter-organelle communication engages also monolayer organelles, such as LDs and RNA granules, or stress granules, which are devoid of membranes [95].

LDs form contact sites with ER, through interaction between acyl-CoA synthetase fatty acid transport protein 1 (FATP1) localized at the ER, and DGAT2, localized at the LD membrane [146].

LD−mitochondria contact sites are established during nutrient deprivation or cell growth, two conditions characterized by a high demand of phospholipids for membrane biosynthesis and lipids for β-oxidation [147,148]. Furthermore, the complex constituted by RAB18 on LDs and the tethering complex NRZ (NAG-RINT1-ZW1) associated with soluble N-ethylmaleimide-sensitive-factor attachment protein receptor (SNAREs) is involved in LD−mitochondria contacts [149].

PLIN5 is localized on the LD membrane, and it was described also as fundamental for LD−mitochondria contact sites for its ability to recruit LDs to mitochondria. Nevertheless, the interacting partner of PLIN5 on the outer mitochondrial membrane is not yet known, and the mechanism of mitochondria recruitment has not been elucidated [150].

Other proteins involved in LD−mitochondria contacts are still under investigation. The interaction between Mfn2 and PLIN1 was identified in LD−mitochondria of brown adipocytes [151]. Moreover, by using the BioID tool that is able to detect candidate protein−protein interactions in living cells, a set of other interactors were identified [152]. For instance, the acyl-CoA synthase long chain family member 1 (ACSL1) on mitochondria interacts with the synaptosomal-associated protein 23 (SNAP23) and vesicle-associated membrane protein 4 (VAMP4) on LDs [153] (Figure 3).

Several studies are ongoing to investigate other proteins involved in LD recruitment on mitochondrial. Moreover, the contacts described so far are highly dynamic, fitting the “kiss and run” paradigm. However, a more stable interaction, known as anchoring, between LDs and mitochondria bind has been described. This type of contact has been identified in oxidative tissues such as brown adipose tissue, skeletal muscle, and the myocardium [154]. Emerging evidence invites questions about the role of LD−mitochondria contacts in immune function. In hepatocytes, lipopolysaccharide induces PLIN 5 downregulation, consequent detachment of mitochondria from LDs, and decrease of β-oxidation and ketogenesis [155]. In contrast, the increase of PLIN5 in human THP-1 macrophages determines increased LD−mitochondria contacts and reduced antibacterial function [155]. Notably, the antibacterial proinflammatory activity of M1 macrophages is based on glycolysis, while anti-inflammatory M2 macrophages preferentially use fatty acid oxidation and oxidative phosphorylation, supporting the rationale for LD−mitochondria detachment in response to M1-polarizing stimuli such as lipopolysaccharide [156].

Aging is characterized by several mitochondrial alterations, from the increase of oxidative stress to alterations in mitochondria dynamics, such as fragmentation of mitochondrial network, reduced number of mitochondria, mtDNA mutagenesis, and loss of mitochondrial membrane potential [157,158]. Recently, in yeast as well as in mammalian cell lines, it was demonstrated that, under stress conditions, the interaction between LDs and mitochondria increased in order to transport toxic proteins from mitochondria to LDs, protecting cells from apoptotic death [159]. Another recent study in yeasts showed that an increase in LD number extended cell survival during the stationary phase, paralleled by a progressive increase in ROS levels. The results obtained in this study suggest that LD−mitochondria contact sites are beneficial for cell fitness during yeast aging. Indeed, LDs contribute to mitochondrial “rejuvenation”, eliminating toxic protein, attenuating oxidative stress, and, finally, indicating a role of LD−mitochondria contact sites in longevity [160].

The transport of molecules between mitochondria and LDs is not limited to proteins, but it also involves lipids. Upon stress condition and aging, phosphatidylinositols and ergosterols (the yeast sterols) increase in mitochondria, while their content decreased in LDs [161]. Proteins and lipids, shuttled between mitochondria and LDs, are then degraded in the lysosome/vacuole during macrolipophagy [162]. Further studies on LD−mitochondria contact sites in mammalian cells are required to confirm the importance of this inter-organelle communication in longevity.

## 7. Mitochondrial Dyshomeostasis and Inflammation in Aging and Related Conditions

A global reduction in the capacity to cope with a variety of stressors and a progressive increase in pro-inflammatory mediators are major characteristics of the aging process [163]. This phenomenon, referred to as “inflamm-aging”, is induced by a continuous antigenic load and stress. Under these conditions, a state of permanent arrest of cell proliferation occurs and leads to cellular senescence characterized by the acquisition of a senescence-associated secretory phenotype (SASP) [164].

The tight relationship between inflammation and cellular senescence has also been shown by gene expression studies revealing inflammatory gene expression patterns similar in the two conditions [165]. Senescent human fibroblasts express several inflammation-associated genes, including monocyte chemotactic protein-1, Gro-α, IL-1β, IL-15, TLR4, and intercellular adhesion molecule 1 [166,167]. Inflammatory genes were found to be up-regulated not only in senescent fibroblasts but also in senescent human hepatic stellate cells [168]. However, this pattern was not found in senescent retinal pigment epithelial cells and vascular endothelial cells, suggesting that inflammatory pathways associated with senescence might be cell-specific.

A set of pro-inflammatory and pro-fibrotic factors and metalloproteases have been identified among SASP molecules [164]. As a consequence of the progressive accumulation of senescent cells and chronically released SASP molecules, inflammation, tissue damage, and fibrosis can eventually ensue, which predispose to age-related conditions (e.g., metabolic disorders, atherosclerosis, muscle wasting and neurodegeneration) [169,170,171]. Tezze et al. [172] found that age-associated loss of the mitochondrial protein optic atrophy 1 (OPA1) was related to muscle atrophy and systemic inflammation. Indeed, OPA1^−/−^ mice showed an early aging phenotype and high circulating levels of several inflammatory cytokines (i.e., IL-1α, IL-1β, IL-6, and TNF-α). The accrual of senescent cells during aging has been described in several animal models [173,174] along with an increase in intracellular damage and reduced senescence immune surveillance [175,176]. A great deal of research is ongoing to evaluate the effects of senolytics/senomorphics at eliminating senescent cells and attenuating SASP production. As such, a deeper understanding of the molecular mechanisms underlying cellular senescence and related signaling routes at the systemic levels is crucial for devising specific anti-aging strategies.

Mitochondrial integrity is preserved by a set of quality control mechanisms of which autophagy, and more specifically mitophagy, plays a major role [77]. Indeed, autophagy limits the accumulation of pro-inflammatory factors and the dysregulation of this process may result in increased cytoplasmic mtDNA-driven inflammation [177]. MtDNA can be degraded by DNAses I contained in the autolysosomes [178], and the mtDNA–TLR9-mediated inflammatory response was found to be increased in the heart of DNase II-deficient mice developing cardiomyopathy [77]. Moreover, sterile inflammation following mitochondrial dysfunction and the concomitant rise in mtDNA levels can trigger the release of IL-1β and IFNα via cGAS engagement [179]. A dysregulation in the clearance of dysfunctional mitochondria has also been hypothesized to determine the escape of oxidized ccf-mtDNA or nucleoids which can trigger inflammation by interacting with PRRs [180]. Moreover, similar to the nuclear DNA system, disruption of processes involved in mtDNA homeostasis, such as mtDNA replication and repair, can trigger cGAS–STING activation. For instance, a defective mtDNA packaging into nucleoids following TFAM depletion is a prominent signal for cGAS activity in diverse cells [181].

Recently, MERCs have also been indicated as molecular platforms contributing to aging and age-related diseases via SASP signaling [182]. Indeed, alterations of MERCs quantity and quality with aging and related diseases have been reported [95,96,104].

Pinti and collaborators [183] described an association between ccf-mtDNA and elevated levels of proinflammatory cytokines during aging. In particular, they observed that the number of copies of ccf-mtDNA increased significantly after the fifth decade of life, peaking past the age of 90 [183]. Interestingly, systemic levels of ccf-mtDNA decrease in healthy adults after moderate aerobic exercise, a well-known anti-inflammatory intervention [184,185,186]. Sliter et al. [187] investigated the effect of exhaustive exercise on inflammation in Parkin^−/−^ or Pink1^−/−^ mice. The authors observed that exhaustive exercise caused a striking increase in serum levels of pro-inflammatory IL-6 and IFN-b. Remarkably, deletion of STING or the administration of IFNAR-blocking antibody completely rescued a normal phenotype, suggesting that mtDNA released from damaged mitochondria that are not cleared is responsible for the observed inflammation [187]. Specific alterations of the mitochondrial quality control axis have also been reported in muscle biopsies from old hip-fractured patients with sarcopenia in whom a link between muscular mitochondrial dysfunction and systemic inflammation via the release of mtDNA has been hypothesized [188].

Inflammation also represents an underlying mechanism of neurodegeneration [189]. Altered mitophagy and activation of cGAS–STING signaling have been indicated as a pathogenic mechanism of Parkinson’s disease [187]. Familial forms of Parkinson’s disease carry missense mutations in PINK1 and Parkin proteins whose deletion in mice induces an accumulation of mtDNA and elevation of systemic cytokine levels [187]. Of note, the latter effect is fully abrogated by co-deletion of STING [187]. The cGAS–STING-dependent inflammation triggered by mtDNA has also been involved in the neuropathological processes associated with amyotrophic lateral sclerosis and frontotemporal lobar degeneration [48]. This has been mainly ascribed to accrual of misplaced mitochondrial of TDP43, a DNA/RNA-binding protein, that induces mtDNA release through mitochondrial transition pore opening and leakage via VDAC1. As a consequence, a release of type I INFs and inflammatory cytokines in a cGAS–STING-dependent manner occurs in human and mouse cells [48]. Depletion of STING in a preclinical model of amyotrophic lateral sclerosis overexpressing TDP43 was also able to dampen neuroinflammation and mitigate disease progression. Finally, the elevation of type I INFs following STING activation has also been shown in models of Huntington disease [190] or neurodegeneration and astrocytic inflammation guided by protein aggregates deposition [191].

## 8. Conclusions

Mitochondrial metabolism and its dysfunction have been listed among the nine pillars of the geroscience paradigm and indicated as key targets amenable for anti-aging interventions. The contribution of age-related changes in inter-organelle contacts to mitochondrial dyshomeostasis has recently been investigated. These molecular platforms are implicated in mitochondrial remodeling and transfer of selected molecules with pro-inflammatory properties. A deeper characterization of these inter-organelle interactions and their contribution to cellular senescence may be very informative towards the intricate pathways regulating cellular decay during aging. The close relationship of mitochondrial signals with cellular senescence and their contribution to aging and age-related conditions confer further priority to investigating these processes.

## Data Availability

No data were generated for the present article.
